# Supplementary material for: Magic in twisted transition metal dichalcogenide bilayers
Source: Nat Commun. 2021 Nov 18;12:6730. doi: 10.1038/s41467-021-27042-9 (PMC8602625; doi:10.1038/s41467-021-27042-9)
Supplement: Supplementary file 1 — Supplementary Information [file 41467_2021_27042_MOESM1_ESM.pdf]

Supplementary Information for "Magic in twisted transition metal dichalcogenide bilayers"

Trithep Devakul, Valentin Crépel, Yang Zhang, and Liang Fu
Department of Physics, Massachusetts Institute of Technology, Cambridge, Massachusetts 02139, USA

I. SUPPLEMENTARY METHODS

A. Supplementary Note 1

Density functional theory calculation and continuum model — We study TMD homobilayers with a small twist angle starting from AA stacking, where every metal (M) or chalcogen (X) atom on the top layer is aligned with the same type of atom on the bottom layer (AB stacking can be viewed as a 180° rotation of top layer). Within a local region of a twisted bilayer, the atom configuration is identical to that of an untwisted bilayer, where one layer is laterally shifted relative to the other layer by a corresponding displacement vector $\boldsymbol{d}_0$. For this reason, the moiré band structures of twisted TMD bilayers can be constructed from a family of untwisted bilayers at various $\boldsymbol{d}_0$, all having 1×1 unit cell. Our analysis thus starts from untwisted bilayers.

In particular, $\boldsymbol{d}_0 = 0, (-\boldsymbol{a}_1 + \boldsymbol{a}_2)/3, (\boldsymbol{a}_1 + \boldsymbol{a}_2)/3$, where $\boldsymbol{a}_{1,2}$ is the primitive lattice vector for untwisted bilayers, correspond to three high-symmetry stacking configurations of untwisted TMD bilayers, which we refer to as MM, XM, MX. In MM (MX) stacking, the M atom on the top layer is locally aligned with the M (X) atom on the bottom layer, likewise for XM. The bilayer structure in these stacking configurations is invariant under three-fold rotation around the z axis.

Density functional calculations are performed using generalized gradient approximation with SCAN+rVV10 Van der Waals density functional, as implemented in the Vienna Ab initio Simulation Package. Pseudopotentials are used to describe the electron-ion interactions. We first construct the zero-twisted angle WSe2/WSe2 bilayer at MM and MX lateral configurations with vacuum spacing larger than 20Å to avoid artificial interaction between the periodic images along the z direction. The structure relaxation is performed with force on each atom less than 0.01 eV/A. We use $12 \times 12 \times 1$ for structure relaxation and self-consistent calculation. The more accurate SCAN+rVV10 van der Waals density functional gives the relaxed layer distances as 6.6Å and 7.15Å for MX and MM stacking structures, respectively. By calculating the work function from electrostatic energy of converged charge density, we plot in Fig. 1 the band structure of MM and MX-stacked bilayers, with reference energy $E = 0$ chosen to be the absolute vacuum level.

To compare with moiré band structure, we further construct the commensurate structures with twist angle 5.08° with 762 atoms, and perform large scale DFT calculation to relax the moiré superlattice. The spacial dependent layer distance profile has been discussed in the main text, and fits nicely with the untwisted MM and MX stacking structures. The moiré band structure with spin orbital coupling agrees remarkably well with the continuum model derived from untwisted calculations, and the effective mass is chosen as $0.38m_e$, close to the experimental value $0.4\sim0.45m_e$. To reveal the real space localization of moiré flat band, the γ point Kohn-Sham wavefunction for the top-most moiré valence band is calculated and projected to bottom and top layer, respectively. As shown in Fig. 2, we find that the charge are localized at MX and XM region, consistent with previous Wannier function.

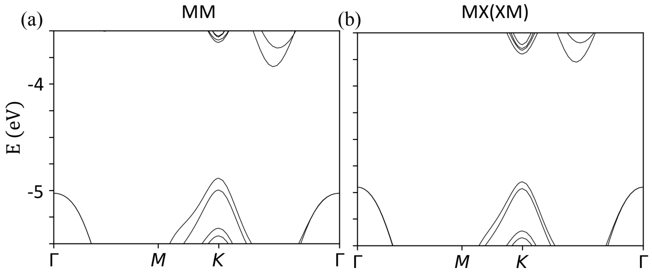

Supplementary Figure 1: Band structure with reference energy E_0 set as the absolute vacuum level in (a) MM stacking structure; (b) MX (XM) stacking structure.

For $MoTe_2$, the Γ pockets appears to be the valence band maximum at MX stacking structure when using lattice constant 3.52Å from bulk structures (The relaxed layer distance is 6.95Å, close to the bulk layer distance 6.98Å in 2H structures). Experimentally, AB stacked bilayer $MoTe_2$ has been shown to be a indirect band gap semiconductor from optical measurements [1, 2]. We thus believe Γ pockets would enter in the moiré valence bands for twisted homobilayer $MoTe_2$ even for AA stacking. At $\theta = 5.08°$, we calculate the moiré band structure of fully relaxed AA stacked homobilayer $MoTe_2$. As shown in Fig. 3, the narrow Dirac like Γ valley moire bands intersect with topmost dispersive K valley moire bands. In transport measurements under zero displacement field, these two type of charge carriers would both enter, causing further complications.

B. Supplementary Note 2

Moiré topology criteria — We determine the Chern number of top two moiré valence bands from their C_{3z}

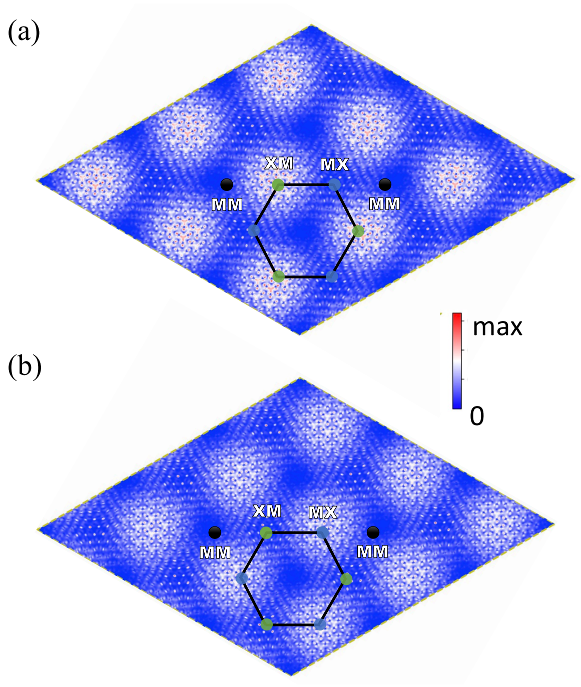

Supplementary Figure 2: γ point Kohn-Sham Wavefunction of the topmost moiré valence band projected at (a) top layer, (b) bottom layer.

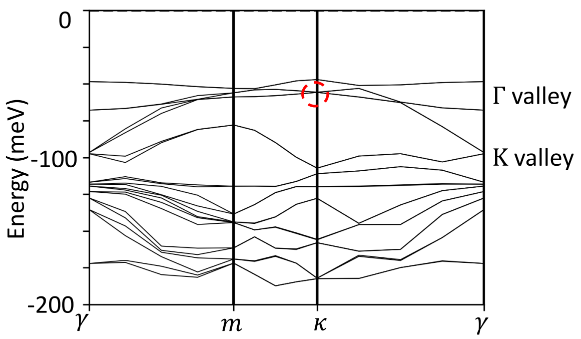

Supplementary Figure 3: Moiré band structure of fully relaxed AA stacked homobilayer $MoTe_2$ at $\theta = 5.08°$. Γ valley moire bands and K valley moire bands are very close in energy, and we circle the Dirac points from Γ valley moire bands.

eigenvalues. Computationally, the Kohn-Sham wavefunctions in plane-wave basis

$$|\Psi_{n\boldsymbol{k}}\rangle = \sum_{\boldsymbol{G}} C_{n\boldsymbol{k}}(\mathbf{G})|\boldsymbol{k}+\boldsymbol{G}\rangle$$

are extracted at three C_{3z} invariant momentum γ, κ, and κ' with energy cutoff as $E_{cut} = 100$ eV and $\hbar^2(\boldsymbol{k}+\boldsymbol{G})^2/2m_e < E_{\mathrm{cut}}$. And we compute their C_{3z} eigenvalue as:

$$\langle\Psi_{nk}|C_{3z}|\Psi_{nk}\rangle = \sum_{\tau\tau'} S_{\sigma\sigma'}(C_{3z}) \left\langle \Psi^{\sigma}_{nk}|C_{3z}|\Psi^{\sigma'}_{nk}\right\rangle \quad (1)$$

where $\sigma, \sigma' = \uparrow$ or $\downarrow$, $\left\langle \Psi^{\sigma}_{nk}|C_{3z}|\Psi^{\sigma'}_{nk}\right\rangle$ calculated from the transformation relation of plane-waves, and $S_{\sigma\sigma'}(C_{3z})$ is the C_{3z} spin rotation matrix.

As there is a valley time reversal symmetry within the moire superlattice, the energy bands between γ, κ and κ' are enforced to be double degenerate from K and $-K$ valley. Therefore, to determine the valley index of moiré bands, we add a small gating field $D = 0.005$ V/nm to introduce a less than 2 meV splitting at κ and κ'. The K and $-K$ indexes are then diagnosed by spin resolved wavefunction. At γ point where bands are still double degenerate with gating field, we extracted the C_{3z} eigenvalues and valley indexes from finite momentum perturbation along γ to κ direction.

C. Supplementary Note 3

Continuum model at higher angles — Figure 4 show the band structure for the continuum model at larger twist angles than discussed in the main text, as well as the band gaps and bandwidth. The bandwidth of the first band W_1 increases rapidly for $\theta > \theta_1 \approx 1.5°$, reaching $W \sim 100$meV for $\theta \sim 5°$–$6°$. The bandwidth of the second band W_2 has a minima at $\theta \approx 2.2°$, and remains quite small throughout the angle range $\theta \sim 2°$–$3°$, with a bandwidth $W_2 \approx 5$meV. Although ε_{12} vanishes at $\theta_2 \approx 3.3°$, ε_{23} remains positive until $\theta \approx 5.4°$. For $1.5° < \theta < 5.4°$, the top two bands are thus separated from the rest of the spectrum and form a combined $\mathcal{C} = 2$ manifold.

D. Supplementary Note 4

Analytic magic angle — In this section, we analytically estimate the magic angle by consider the effective mass of the top band near γ.

At $k = \gamma$, when $w = V = 0$, the highest energy states are sixfold degenerate plane wave states at energy $e_0 = -\frac{|\gamma-\kappa_\pm|^2}{2m^*} = -\frac{8\pi^2\theta^2}{9m^* a_0^2}$, which is separated from the next set of eigenstates at $e_1 = -\frac{32\pi^2\theta^2}{9m^* a_0^2}$. Incorporating the effect of w and V will introduce coupling between these states at e_0, as well as with those at e_1 and beyond. For the purposes obtaining an analytic estimate for the magic angle, we include only this set of 6 highest energy states. This approximation is valid for $w, V \ll e_0 - e_1$: as a rough estimate of the validity, $e_0 - e_1 \approx 130$meV at $\theta = 1.5°$ is an order of magnitude greater than $(V, w) = (9, 18)$meV for WSe_2.

These six states consist of the plane waves at $\boldsymbol{k}_j$ for $j = 1 \dots 6$, where

$$\boldsymbol{k}_j = \begin{cases} \boldsymbol{\kappa}_+ + (\mathcal{R}_{\pi/3})^{j-1}\boldsymbol{\delta} & \text{if } j \text{ odd} \\ \boldsymbol{\kappa}_- + (\mathcal{R}_{\pi/3})^{j-1}\boldsymbol{\delta} & \text{if } j \text{ even} \end{cases} \quad (2)$$

and the j odd (even) states are on the top (bottom) layer, which we label by $|j\rangle$. Here, $\boldsymbol{\delta} = [2\pi\theta/(3a_0)](\sqrt{3}, -1)$

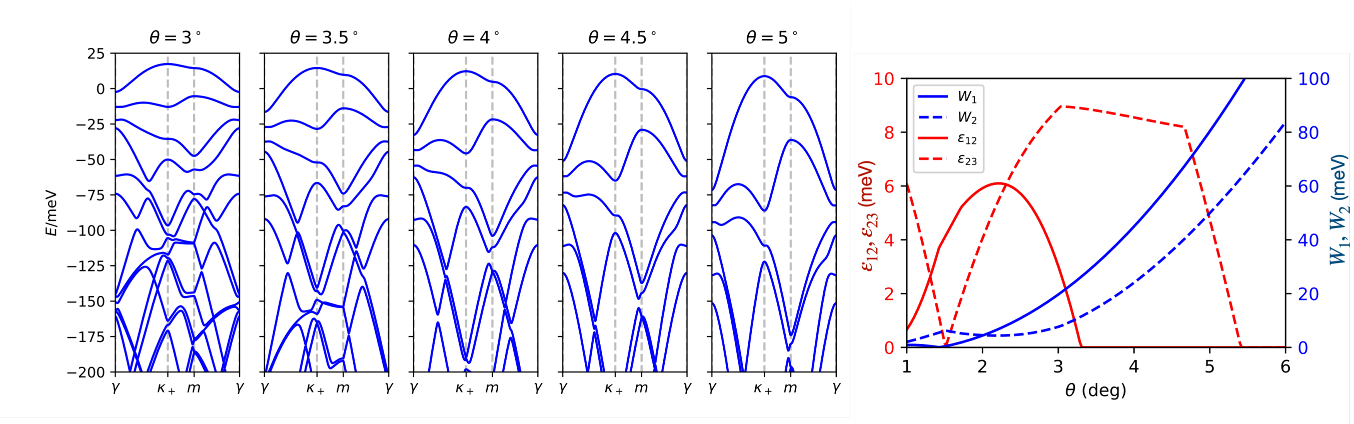

Supplementary Figure 4: (left) Band structure of the continuum model at twist angles $\theta = 3°$–$5°$, larger than shown in the main text. (right) The bandwidth W_1, W_2 of the first and second bands and the indirect energy gaps between the first two bands, ε_{12}, and between the second and third bands ε_{23}.

and R_θ is a θ counter-clockwise rotation matrix. The continuum Hamiltonian acts on these states as

$$\mathcal{H}_\uparrow |j\rangle = e_0 |j\rangle + V\left(e^{i\psi}|j+2\rangle + e^{-i\psi}|j-2\rangle\right) + w\left(|j+1\rangle + |j-1\rangle\right) + \cdots \tag{3}$$

where $|j \pm 6\rangle \equiv |j\rangle$, and $\cdots$ contain states outside of $\{|j\rangle\}$, which we have ignored due to the large energy gap $e_1 - e_0$. Utilizing the emergent C_6 symmetry which sends $|j\rangle \to |j+1\rangle$, $\mathcal{H}_\uparrow$ is easily diagonalized (in the restricted subspace) by eigenstates

$$|n\rangle = \frac{1}{\sqrt{6}} \sum_{j=1}^{6} e^{in\pi j/3} |j\rangle \tag{4}$$

with eigenvalue $e_0 + \mathcal{E}_n$, where

$$\mathcal{E}_n = 2w\cos(\pi n/3) + 2V\cos(2\pi n/3 - \psi). \tag{5}$$

In the absence of any accidental degeneracy, there is a unique highest energy eigenstate $|n_0\rangle$.

Next, in order to estimate when $\boldsymbol{\gamma}$ transitions from band maxima to minima, we consider a small momentum shift about $\boldsymbol{\gamma}$. The C_3 symmetry of the microscopic model restricts the lowest order momentum dependence to be simply $\frac{1}{2m_\gamma}|\boldsymbol{k} - \boldsymbol{\gamma}|^2$ for some effective mass m_γ. Thus, we consider a momentum shift $\epsilon\hat{x}$ away from $\boldsymbol{\gamma}$ and work perturbatively in ϵ. Expanding the energy $E = E^{(0)} + \epsilon^2 E^{(2)}$, we have that $E^{(2)} = 1/(2m_\gamma)$, and the magic angle $\tilde{\theta}_m$ is where $E^{(2)}$ changes sign.

We expand $\mathcal{H}_\uparrow$ as $\mathcal{H}_\uparrow = H_0 + \epsilon H_1 + \epsilon^2 H_2$, which act on $|j\rangle$ as

$$H_1 |j\rangle = -\frac{\hat{x}\cdot(\mathcal{R}_{\pi/3})^{j-1}\boldsymbol{\delta}}{m^*} |j\rangle \tag{6}$$

and $H_2 = -\frac{1}{2m^*}$. We have

$$\hat{x}\cdot(\mathcal{R}_{\pi/3})^{j-1}\boldsymbol{\delta} = \frac{4\pi\theta}{3a_0}\sin(j\pi/3) \tag{7}$$

hence, acting on $|n\rangle$,

$$H_1 |n\rangle = \frac{2\pi\theta}{3m^* a_0}\left(|n+1\rangle + |n-1\rangle\right). \tag{8}$$

Applying standard perturbation theory in the $|n\rangle$ basis, the second order correction to the energy is

$$E^{(2)} = \langle n_0|H_2|n_0\rangle + \sum_{n\neq n_0} \frac{\langle n_0|H_1|n\rangle\langle n|H_1|n_0\rangle}{\mathcal{E}_{n_0} - \mathcal{E}_n} = -\frac{1}{2m^*} + \frac{4\pi^2\theta^2}{9m^{*2}a_0^2}\left(\frac{1}{\mathcal{E}_{n_0} - \mathcal{E}_{n_0+1}} + \frac{1}{\mathcal{E}_{n_0} - \mathcal{E}_{n_0-1}}\right) \tag{9}$$

Solving for where $E^{(2)} = 0$ gives the magic angle condition from the main text,

$$\tilde{\theta}_m^{-2} = \frac{8\pi^2}{9m^* a_0^2}\left(\frac{1}{\mathcal{E}_{n_0} - \mathcal{E}_{n_0+1}} + \frac{1}{\mathcal{E}_{n_0} - \mathcal{E}_{n_0-1}}\right) \tag{10}$$

E. Supplementary Note 5

Localized Wannier functions and tight binding model — In this section, we provide additional details on the construction of the Wannier states shown in the main text. We numerically diagonalize the Hamiltonian $\mathcal{H}_\uparrow$ on an $N_k = 30\times 30$ discretization of the moiré Brillouin zone, corresponding to a system of 30×30 moiré unit cells. For each $\boldsymbol{k}$, the eigenstates $\{|\phi_{n,\boldsymbol{k}}\rangle\}_n$ is obtained in the plane wave basis, expressed as $|\phi_{n\boldsymbol{k}}\rangle = \sum_{\boldsymbol{g},l} c_{\boldsymbol{g}l}^{(n\boldsymbol{k})} |\boldsymbol{k}+\boldsymbol{g}, l\rangle$, where $\boldsymbol{g}$ are reciprocal moiré lattice vectors, $l = \pm$ is the layer, and $c_{\boldsymbol{g}l}^{(n\boldsymbol{k})}$ are complex numbers obtained from exact diagonalization. The diagonalization is done by truncating to some maximum $|\boldsymbol{k}+\boldsymbol{g}|$ which is sufficiently far from the valence band top at $\boldsymbol{\kappa}_\pm$ so as to have negligible effect on the first few moiré bands.

Constructing Wannier states for the top two bands involves finding the appropriate 2×2 unitary matrices $U_{nm}^{(\boldsymbol{k})}$

at each $\boldsymbol{k}$. For convenience, we shall suppress all $\boldsymbol{k}$ labels. We parameterize the unitary matrix as

$$U = \begin{pmatrix} e^{i\psi_1}\cos(\chi/2) & e^{i\psi_1+i\delta}\sin(\chi/2) \\ e^{i\psi_2-i\delta}\sin(\chi/2) & -e^{i\psi_2}\cos(\chi/2) \end{pmatrix}, \tag{11}$$

where $\chi \in [0,\pi]$, and $\delta, \psi_1, \psi_2 \in [0, 2\pi]$. As stated in the main text, U is chosen to maximize the layer polarization of $|\tilde{\phi}_n\rangle = \sum_m U_{nm} |\phi_m\rangle$,

$$P = \sum_{n=1,2} (-1)^n \langle \tilde{\phi}_n | (\mathcal{P}_- - \mathcal{P}_+) | \tilde{\phi}_n \rangle, \tag{12}$$

where $\mathcal{P}_\pm$ projects to the $\pm$ layer. Using $\mathcal{P}_- = 1 - \mathcal{P}_+$, this can be simplified to

$$\begin{aligned} P &= -2 \sum_{n=1,2} (-1)^n \langle \tilde{\phi}_n | \mathcal{P}_+ | \tilde{\phi}_n \rangle \\ &= -2 \sum_{n,m,l} (-1)^n \langle \phi_m | U^\dagger_{mn} \mathcal{P}_+ U_{nl} | \phi_l \rangle \\ &= 2\left[\cos(\chi)(P_{11} - P_{22}) + \sin(\chi)(e^{i\delta} P_{12} + e^{-i\delta} P_{21})\right] \end{aligned} \tag{13}$$

where $P_{nm} = \langle \phi_n | \mathcal{P}_+ | \phi_m \rangle = P^*_{mn}$.

It is straightforward to maximize P with respect to χ and δ. First, setting $\partial_\delta P = 0$ gives $\delta = \arg P_{21} + s\pi$, where $s \in \{0,1\}$. Then, setting $\partial_\chi P = 0$ gives

$$\chi_s = \arctan\left(\frac{2(-1)^s |P_{12}|}{P_{11} - P_{22}}\right) \in [0,\pi] \tag{14}$$

(notice the range of arctan is taken to be $[0,\pi]$). The two solutions for $s = 0,1$ correspond to the maximum and minimum of P. To find the maximum, we simply compare the value of P for these two solutions and take the larger one.

To determine the phase factor $\psi_{1,2}$, we take the real space component of the wavefunction at the MX/XM stacking regions. Define $\boldsymbol{r}_1 = (a_0/2\theta)[-1/\sqrt{3}, 1]$ and $\boldsymbol{r}_2 = (a_0/2\theta)[1/\sqrt{3}, 1]$ to be the locations of the XM and MX stacking region. We then take the phase factors $\psi_{1,2}$ such that $\langle \boldsymbol{r}_n, l_n | \tilde{\phi}_1 \rangle$ is real and positive, where $l_{1(2)} = +(-)$ (here, $|\boldsymbol{r}, l\rangle$ is the usual real space state at $\boldsymbol{r}$ on layer l, satisfying $\langle \boldsymbol{r}, l | \boldsymbol{k} + \boldsymbol{g}, l' \rangle = e^{i(\boldsymbol{k}+\boldsymbol{g})\cdot \boldsymbol{r}} \delta_{ll'}$). This fixes the phase factors $\psi_n = -\arg \langle \boldsymbol{r}_n, l_n | \tilde{\phi}_n \rangle$.

Finally, the Wannier functions localized at $\boldsymbol{R} + \boldsymbol{r}_n$, for a moiré lattice vector $\boldsymbol{R}$, are defined

$$|W^n_{\boldsymbol{R}}\rangle = \frac{1}{\sqrt{N_k}} \sum_{\boldsymbol{k}} e^{-i\boldsymbol{k}\cdot\boldsymbol{R}} |\tilde{\phi}_{n,\boldsymbol{k}}\rangle. \tag{15}$$

The Wannier centers $\boldsymbol{R} + \boldsymbol{r}_n$ form a moiré honeycomb lattice on the XM and MX stacking regions.

The hopping matrix elements between Wannier states on the site $\boldsymbol{R} + \boldsymbol{r}_n$ and $\boldsymbol{R}' + \boldsymbol{r}_{n'}$ can be obtained from the continuum Hamiltonian and U matrices. We have

$$\langle W^{n'}_{\boldsymbol{R}'} | \mathcal{H}_\uparrow | W^n_{\boldsymbol{R}} \rangle = \frac{1}{N_k} \sum_{\boldsymbol{k}} e^{i\boldsymbol{k}\cdot(\boldsymbol{R}'-\boldsymbol{R})} \sum_m U^{(\boldsymbol{k})\dagger}_{mn'} E_m(\boldsymbol{k}) U^{(\boldsymbol{k})}_{nm} \tag{16}$$

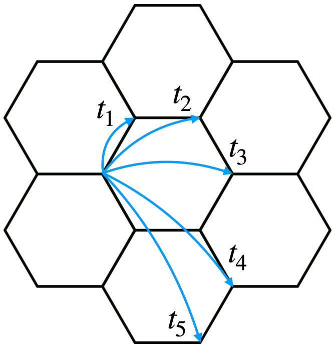

Supplementary Figure 5: Illustration of the hopping matrix elements t_n in the effective tight binding model for spin-$\uparrow$ electrons.

where $E_m(\boldsymbol{k})$ is the energy of $|\phi_{m\boldsymbol{k}}\rangle$ under $\mathcal{H}_\uparrow$. Using this method, we compute the hopping matrix elements between nth nearest neighbor sites up to $n = 5$ as illustrated in Fig 5, and shown in the main text. We find that t_n are mostly real with the exception of t_2, which we define (see Fig 5) to be the 2nd nearest neighbor hopping term along right-turning path. From the symmetries of the model, it follows that the hopping term along a left-turning path is t_2^*. The $-K$ valley produces a similar tight binding model of spin-$\downarrow$ electrons related to this one by time reversal symmetry. As a final remark, we note that the Wannier states obtained through this method are not maximally localized, thus our tight binding model has the potential to be further improved.

F. Supplementary Note 6

Effective Spin Hamiltonian — The derivation of an effective spin Hamiltonian from a perturbative expansion of Eq. 6 in the limit $|t_1|, |t_2| \ll \Delta, U$ is rather complicated when no clear scale separation exists between Δ and U. Fortunately in our model, the interaction scale U largely dominates over the tunneling terms of $\mathcal{H}_{\mathrm{TB}}$, and all experimentally relevant applied displacement fields Δ. As a consequence, we may first eliminate all doubly occupied configurations, and then use $t_1 \ll \Delta$ to obtain the effective spin Hamiltonian given in the main text.

1. *Strong coupling expansion*

Let us first fix some notations. We write the interacting Kane-Mele model as

$$\mathcal{H}_{\mathrm{tot}} = \mathcal{H}_U + \mathcal{T}_- + \mathcal{T}_0 + \mathcal{T}_+, \tag{17}$$

where $\mathcal{T}_u$ gathers all single-particle terms which change the number of doubly occupied by u. To get their explicit expression, we gather in $T^{(1)}_{\delta,u}$ the nearest neighbor tunneling processes changing the number of occupied A site

by δ and the number of doubly occupied sites by u, where $\delta = \pm$ and $u = 0, \pm$. A similar definition $T^{(2)}_{0,u}$ applies for next-nearest neighbor hoppings. These definition yield

$$\mathcal{T}_0 = \mathcal{H}_\Delta + T^{(2)}_{0,0} + T^{(1)}_{+,0} + T^{(1)}_{-,0}, \tag{18a}$$

$$\mathcal{T}_\pm = T^{(2)}_{0,\pm} + T^{(1)}_{+,\pm} + T^{(1)}_{-,\pm}. \tag{18b}$$

Elimination of doubly occupied configurations is achieved by standard perturbation theory techniques, see for instance Ref. [3], and gives

$$\begin{aligned} \mathcal{H}^U_{\text{tot}} &= \mathcal{T}_0 - \frac{1}{U}\mathcal{T}_-\mathcal{T}_+ \\ &= \mathcal{H}_\Delta + \tilde{\mathcal{T}}_{-2} + \tilde{\mathcal{T}}_{-1} + \tilde{\mathcal{T}}_0 + \tilde{\mathcal{T}}_{+1} + \tilde{\mathcal{T}}_{+2}, \end{aligned} \tag{19}$$

that we have recast in a form more amenable to perturbative expansion in Δ by introducing

$$\begin{aligned} \tilde{\mathcal{T}}_0 &= T^{(2)}_{0,0} - \frac{1}{U}\left[T^{(2)}_{0,-}T^{(2)}_{0,+} + T^{(1)}_{+,-}T^{(1)}_{-,+} + T^{(1)}_{-,-}T^{(1)}_{+,+}\right], \\ \tilde{\mathcal{T}}_{\pm1} &= T^{(1)}_{\pm,0} - \frac{1}{U}\left[T^{(2)}_{0,-}T^{(1)}_{\pm,+} + T^{(1)}_{\pm,-}T^{(2)}_{0,+}\right], \\ \tilde{\mathcal{T}}_{\pm2} &= -\frac{1}{U}T^{(1)}_{\pm,-}T^{(1)}_{\pm,+}. \end{aligned} \tag{20}$$

Similar to the previous case, $\tilde{\mathcal{T}}_\delta$ changes the number of occupied A sites by δ. When their potential energy is large $t_1 \ll \Delta$, these A-sites can be eliminated in a similar way as before, see Ref. [4] for details. This leads to the following effective Hamiltonian in the physical subspace with no double occupations and no occupied A sites:

$$\begin{aligned} \mathcal{H}^{U\Delta}_{\text{tot}} &= \tilde{\mathcal{T}}_0 - \frac{1}{\Delta}\tilde{\mathcal{T}}_{-1}\tilde{\mathcal{T}}_{+1} - \frac{1}{2\Delta}\tilde{\mathcal{T}}_{-2}\tilde{\mathcal{T}}_{+2} \\ &+ \frac{1}{8\Delta^2}\Big[8\tilde{\mathcal{T}}_{-1}\tilde{\mathcal{T}}_0\tilde{\mathcal{T}}_{+1} + 4\left(\tilde{\mathcal{T}}_{-1}\tilde{\mathcal{T}}_{-1}\tilde{\mathcal{T}}_{+2} + \tilde{\mathcal{T}}_{-2}\tilde{\mathcal{T}}_{+1}\tilde{\mathcal{T}}_{+1}\right) \\ &\quad - 4\left(\tilde{\mathcal{T}}_{-1}\tilde{\mathcal{T}}_{+1}\tilde{\mathcal{T}}_0 + \tilde{\mathcal{T}}_0\tilde{\mathcal{T}}_{-1}\tilde{\mathcal{T}}_{+1}\right) + 2\tilde{\mathcal{T}}_{-2}\tilde{\mathcal{T}}_0\tilde{\mathcal{T}}_{+2} \\ &\quad - \left(\tilde{\mathcal{T}}_{-2}\tilde{\mathcal{T}}_{+2}\tilde{\mathcal{T}}_0 + \tilde{\mathcal{T}}_0\tilde{\mathcal{T}}_{-2}\tilde{\mathcal{T}}_{+2}\right)\Big] + \cdots \end{aligned} \tag{21}$$

This expression can be greatly simplified by noticing that the direct action of $T^{(1)}_{\pm,\pm}$ and $T^{(2)}_{0,0}$ is zero on the physical space with no double occupancy and no occupied A sites. Furthermore, all terms of order U^{-2} should be discarded for consistency with our first expansion Eq. 19. Using these insights, we obtain

$$\begin{aligned} \mathcal{H}^{U\Delta}_{\text{tot}} &= -\frac{1}{U}T^{(2)}_{0,-}T^{(2)}_{0,+} - \frac{1}{\Delta}T^{(1)}_{-,0}T^{(1)}_{+,0} + \frac{1}{\Delta^2}T^{(1)}_{-,0}T^{(2)}_{0,0}T^{(1)}_{+,0} \\ &+ \frac{1}{U\Delta}\left[T^{(1)}_{-,0}T^{(1)}_{+,-}T^{(2)}_{0,+} + T^{(2)}_{0,-}T^{(1)}_{-,+}T^{(1)}_{+,0}\right] \\ &- \frac{1}{U\Delta^2}T^{(1)}_{-,0}T^{(1)}_{+,-}T^{(1)}_{-,+}T^{(1)}_{+,0} + P \end{aligned} \tag{22}$$

where we have isolated the contribution

$$P = \frac{1}{U\Delta^2}\left[\frac{T^{(1)}_{-,0}T^{(1)}_{+,0}T^{(2)}_{0,-}T^{(2)}_{0,+}}{2} - T^{(1)}_{-,0}T^{(2)}_{0,0}T^{(1)}_{+,-}T^{(2)}_{0,+} + hc\right] \tag{23}$$

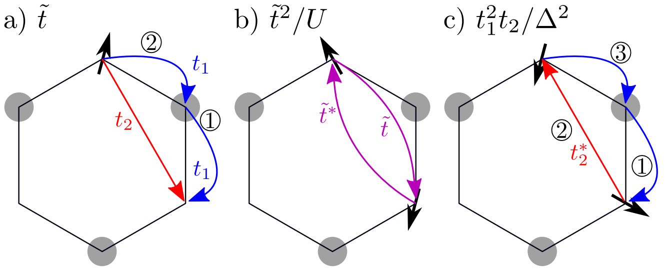

Supplementary Figure 6: Multi-tunneling processes appearing in the perturbation expansion for large displacement field and large interaction scale. a) Effective tunneling $\tilde{t} = t_2 + t_1^2/\Delta$ on the B triangular lattice after elimination of the A site with large potential energy. b) Spin exchange $\propto \tilde{t}^2/U$ between localized spins. c) Additional loop exchange $\propto t_1^2 t_2/U$ process which does not involve any double occupations. The choice of t_2/t_2^* in the diagrams applies to spin-up electrons. Grey circles denote doubly occupied B sites, while arrows represents the spin of the holes on A sites.

that we from now on neglect since its magnitude $|t_1 t_2|^2/(U\Delta^2)$ is much smaller than the other terms appearing in $\mathcal{H}^{U\Delta}_{\text{tot}}$ when $|t_2| \ll |t_1|$, as is the case for our effective tight-binding model (see I E).

Discarding an overall energy shift, we can rewrite the Hamiltonian derived above as

$$\begin{aligned} \mathcal{H}_S = &-\frac{1}{U}\left[T^{(2)}_{0,-} - \frac{1}{\Delta}T^{(1)}_{-,0}T^{(1)}_{+,-}\right]\left[T^{(2)}_{0,+} - \frac{1}{\Delta}T^{(1)}_{-,+}T^{(1)}_{+,0}\right] \\ &+ \frac{1}{\Delta^2}T^{(1)}_{-,0}T^{(2)}_{0,0}T^{(1)}_{+,0}. \end{aligned} \tag{24}$$

The first line simply describes the virtual occupation of a doubly excited state using the bare or an effective next-nearest tunneling, while the second line corresponds to a particle exchange process where particle turn around a small loop of the honeycomb lattice to avoid double occupations. They are sketched in Fig. 6.

It is worth noting here that the terms obtained in $\mathcal{H}_S$ are simply those one would have guessed without decoupling the U and Δ expansions. This is however only due to the facts that the operators $\tilde{\mathcal{T}}_{\pm2}$ are zero in the physical space considered in our model. This is why we have preferred the more lengthy, but more reliable, method presented here.

2. *Rewriting with spin operators*

Introducing the effective tunneling $\tilde{t} = t_2 + t_1^2/\Delta$, we can rewrite the first line of Eq. 24 as

$$\frac{4}{U}\sum_{\langle i,j\rangle_B} |\tilde{t}|^2 s^z_i s^z_j + \text{Re}\,(\tilde{t}^2)(s^x_i s^x_j + s^y_i s^y_j) + \text{Im}\,(\tilde{t}^2)(\boldsymbol{s}_i \times \boldsymbol{s}_j)\cdot\boldsymbol{z} \tag{25}$$

which corresponds to the processes sketched in Fig. 6a and 6b. With a similar calculation, we can recast the second line as

$$\frac{4t_1^2}{\Delta^2}\sum_{\langle i,j\rangle_B}\mathrm{Re}\,(t_2)\boldsymbol{s}_i\cdot\boldsymbol{s}_j+\mathrm{Im}\,(t_2)(\boldsymbol{s}_i\times\boldsymbol{s}_j)\cdot\boldsymbol{z}, \tag{26}$$

which is sketched in Fig. 6c. Putting everything together, we obtain an XXZ model with Dzyaloshinskii–Moriya interactions for the spin localized on the triangular B lattice:

$$\mathcal{H}_S=\sum_{\langle i,j\rangle_B}J_{\|}s_i^z s_j^z+J_\perp(s_i^x s_j^x+s_i^y s_j^y)+D\left[(\boldsymbol{s}_i\times\boldsymbol{s}_j)\cdot\boldsymbol{z}\right], \tag{27}$$

where the coefficients read

$$J_{\|}=\frac{4|\tilde{t}|^2}{U}+\mathrm{Re}\left(\frac{4t_1^2t_2}{\Delta^2}\right), \tag{28a}$$

$$J_\perp=\mathrm{Re}\left(\frac{4\tilde{t}^2}{U}+\frac{4t_1^2t_2}{\Delta^2}\right), \tag{28b}$$

$$D=\mathrm{Im}\left(\frac{4\tilde{t}^2}{U}+\frac{4t_1^2t_2}{\Delta^2}\right). \tag{28c}$$

This is the form used in the main text.

3. Classical phase diagram

The phases appearing for large displacement fields in our Hartree-Fock study (Fig. 5 in the main text) can be rationalized by studying the classical version of Eq. 27. Treating the spins as classical vectors of fixed lengths and imposing the 'weak constraint' of Luttinger-Tisza [5], the spin model can be exactly solved in momentum space.

We find spin eigenstates with spin polarized along $\boldsymbol{z}$ with the dispersion

$$E_z(\boldsymbol{k})=J_{\|}C(\boldsymbol{k}), \tag{29}$$

and two other with spins pined in the xy plane

$$E_{xy}^{\pm}(\boldsymbol{k})=J_\perp C(\boldsymbol{k})\pm|DS(\boldsymbol{k})|, \tag{30}$$

where we have introduced the two auxiliary functions

$$C(\boldsymbol{k})=\sum_{j=1}^{3}\cos(\boldsymbol{k}\cdot\boldsymbol{a}_j),\quad S(\boldsymbol{k})=\sum_{j=1}^{3}\sin(\boldsymbol{k}\cdot\boldsymbol{a}_j), \tag{31}$$

with $\boldsymbol{a}_3=-(\boldsymbol{a}_1+\boldsymbol{a}_2)$. The competition between the out of plane and in-plane modes was studied beyond the classical approximation with the ED spin-wave analysis (see main text). Thus, we discard the E_z eigenstates and look for spin patterns that minimize $E_{xy}^{\pm}$.

Depending on $J_\perp$ and D, the minimum of E_{xy}^{-} is either at the γ point or at $\kappa_\pm$, which respectively describe the FM_{xy} and AFM_{xy} phases of the main text, after reintroduction of Luttinger-Tisza's 'strong constraint' [5]. Comparison of the energies at these three momenta give the conditions

$$\begin{cases}\mathrm{FM}_{xy}: & |D|+\sqrt{3}J_\perp<0,\\ \mathrm{AFM}_{xy}: & |D|+\sqrt{3}J_\perp>0.\end{cases} \tag{32}$$

[1] I. G. Lezama, A. Arora, A. Ubaldini, C. Barreteau, E. Giannini, M. Potemski, and A. F. Morpurgo, Nano letters **15**, 2336 (2015).
[2] C. Ruppert, O. B. Aslan, and T. F. Heinz, Nano letters **14**, 6231 (2014).
[3] J. Stein, Journal of Statistical Physics **88**, 487 (1997).
[4] C. Knetter and G. S. Uhrig, The European Physical Journal B-Condensed Matter and Complex Systems **13**, 209 (2000).
[5] J. M. Luttinger and L. Tisza, Phys. Rev. **70**, 954 (1946).
